# Supplementary material for: Investigating the potential neurotoxic effects of cell-free factors by Batrachochytrium dendrobatidis on locomotion in Xenopus laevis
Source: Biol Open. 2026 Jan 26;15(1):bio062325. doi: 10.1242/bio.062325 (PMC12893034; doi:10.1242/bio.062325)
Supplement: Supplementary information [file biolopen-15-062325-s1.pdf]

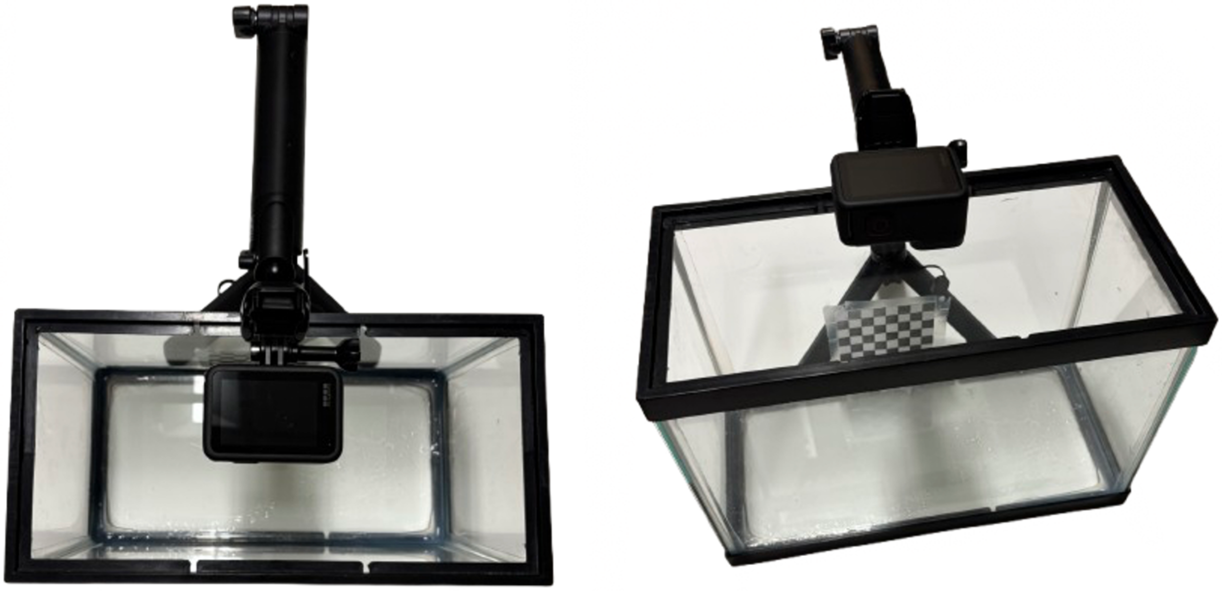

Fig. S1. Both a dorsal and anterolateral view of the tank setup is show below. A 1x1 centimeter grid was placed on the posterior and underside of the tank, though water level was maintained at a height such that frogs could only swim in the horizontal plane.

Biology Open • Supplementary information
